# Flavor Changes of *Tricholoma matsutake* Singer under Different Processing Conditions by Using HS-GC-IMS

**DOI:** 10.3390/foods10030531

**Published:** 2021-03-04

**Authors:** Mengqi Li, Hanting Du, Songyi Lin

**Affiliations:** National Engineering Research Center of Seafood, School of Food Science and Technology, Dalian Polytechnic University, Dalian 116034, China; lmqi912@163.com (M.L.); Duhanting960415@163.com (H.D.)

**Keywords:** *Tricholoma matsutake* Singer, headspace-gas chromatography-ion mobility spectrometry (HS-GC-IMS), principal component analysis (PCA), volatile compounds, characteristic volatile fingerprinting

## Abstract

Headspace-gas chromatography-ion mobility spectrometry (HS-GC-IMS) was used to analyze the changes to volatile compounds in fried *Tricholoma matsutake* Singer under different heating temperatures and times. A total of 40 signals that corresponded to 24 compounds were identified through this approach. Differences in volatile compounds of *T. matsutake* samples were shown in topographic plots and fingerprints. The heating temperatures were more important than the heating times in affecting the volatile compounds. Moreover, changes to the main volatile compounds in *T. matsutake* under different processing conditions were based on the thermal decomposition and a series of chemical reactions of C8 compounds. Principal component analysis (PCA) results showed that samples under different processing conditions could be effectively distinguished. Hence, the combination of HS-GC-IMS and PCA can identify and classify the volatile compounds of *T. matsutake* quickly and sensitively. This study provides a new supplementary analytical method for the rapid identification of T. matsutake. The above results can provide a theoretical basis for the quality control and change mechanism of flavor in the processing of traditional edible fungi products.

## 1. Introduction

*Tricholoma matsutake* Singer (pine mushroom) is one of the rarest and most precious natural edible fungi in the world, with a unique flavor and smooth taste that is becoming more and more popular among consumers around the world. It is rich in proteins, amino acids, unsaturated fatty acids, vitamins, and dietary fiber, with a low fat content, and contains various bioactive ingredients with high medicinal and nutritional value; it is an excellent source of nutrients [1,2,3]. In addition, *T. matsutake* is generally regarded as a high-value food that can not only supplement the body with comprehensive nutrients, but also enhance the body’s immunity, preventing and suppressing the occurrence of many chronic diseases. Previous studies have reported that ergosterols, polysaccharides, polyphenols, and other bioactive substances in *T. matsutake* have the functions of enhancing immunity, anti-oxidation, anti-tumor, anti-bacterial, anti-mutation, and anti-radiation [4,5,6].

In recent years, *T. matsutake* has not only been used as nutritional basic material or food flavoring material but also has been added to food as a single and compounding prescription for the prevention and treatment of diseases for medicinal or functional purposes, and has become a highly demanded product for consumers worldwide [7]. Flavor is an important factor in determining whether certain foods will be accepted by consumers [8]. With the increasing emphasis on a healthy diet, the balance between nutritional value and flavor characteristics of food becomes critically important [9]. Therefore, a detailed understanding of the flavor characteristics of different foods is the key to developing better foods and ultimately satisfying consumer preferences. Guo et al. [10] evaluated the different volatile compounds in the lower stipe, upper stipe, and pileus of fresh and dried T. matsutake. Li et al. [11] reported a method for developing flavor fingerprints for the volatile compounds. However, less information is available on the analysis of the flavor changes in fried *T. matsutake* samples under different processing conditions.

Headspace-gas chromatography-ion mobility spectrometry (HS-GC-IMS) is a simple, rapid, and sensitive analytical technique found in recent years for the detection of volatile compounds in a mixture of analytes [12]. HS-GC-IMS can detect volatile compounds in liquid or solid samples and establish fingerprints without any related pretreatment. It can also treat a large number of samples in a short time and detect volatile compounds at the ppb level in real time. The temporary characterization of volatile compounds based on HS-GC-IMS consists of GC retention and drift time [13]. This technique investigates reliable and unbiased information from a large amount of data through non-target analysis, which plays an important role in the process of screening specific makers. Therefore, HS-GC-IMS technology has been widely applied in a variety of fields, such as pharmaceuticals, security, food science (honey, olive oil, goat cheese, Iberian ham, and egg), and other chemical contaminants [14,15,16,17].

With the development of the food industry, the deep processing of traditional edible fungi has received more and more attention. As an ongoing study, the objective of this work was to identify the differentiation and possible pathways of flavor changes in *T. matsutake* samples under different processing conditions. Principal component analysis (PCA) was applied to characterize the fried *T. matsutake* samples based on the HS-GC-IMS data. These may provide a theoretical basis for the quality control and change mechanism of flavor in the processing of traditional edible fungi products.

## 2. Material and Methods

### 2.1. Materials

T. matsutake samples were purchased from Shunxin Matsutake Trading Co., Ltd. in Yanji City and produced in Changbai Mountain, Jilin Province. After removing the surface dirt, the *T. matsutake* samples were lengthwise cut into uniform slices of about 5 mm thickness and then stored in polyethylene bags at −80 °C for subsequent analyses. Soybean oil used for frying in this experiment was purchased from a local supermarket.

### 2.2. Preparation of Fried T. Matsutake

Each batch of *T. matsutake* slices was fried in 1 L of soybean oil using a deep-frying pan (Guangzhou Aishqi Electrical Technology Co., Ltd., Guangzhou, China). Each frying was done in triplicate by using 50 gm of *T. matsutake* slices for each frying time. When the frying time was 80 s, the frying temperatures were 100, 120, 160, and 200 °C. When the frying temperature was 160 °C, the frying time was 40, 60, 80, 100, and 120 s. The treated samples were cooled with ice, and the thoroughly cooled samples were stored at −80 °C for the subsequent analyses.

### 2.3. HS-GC-IMS Analysis

Analysis of the fried *T. matsutake* samples was performed on a GC-IMS device (FlavourSpec^®^, Gesellschaft für Analytische Sensorsysteme GmbH, Dortmund, Germany). The device was equipped with an automatic sampler unit (CTC Analytics AG, Zwingen, Switzerland). For analysis, the samples (the same samples mentioned in Section 2.1 and Section 2.2) were treated with micronized WK-1000A (Zhongxi Yuanda Technology Co., Ltd., Beijing, China). A total of 0.3 g fine powder of each *T. matsutake* sample was weighed and placed in 20 mL headspace glass extraction vials. The samples were then placed in the GC-IMS instrument and incubated at 40 °C for 25 min. After incubation, 500 µL of the sample headspace was automatically injected into the injector (80 °C, splitless mode) by a heated syringe at 50 °C.

With the aids of carrier gas (nitrogen with a purity of 99.999%), the samples were subsequently pushed into an FS-SE-54-CB capillary column, which had been heated to 40 °C isothermal conditions. The nitrogen flow rate procedure was as follows: 2 mL/min for 2 min, 30 mL/min for 8 min, 100 mL/min for 10 min, and 150 mL/min for 5 min. The analytes were eluted into the ionization chamber and ionized in a positive ion mode by a 3H ionization source with 300 MBq activity. A tritium source (6.5 keV) was used to ionize the molecules. The resulting ions were driven to a drift tube (9.8 cm length) that was operated under conditions of a constant temperature supply of 45 °C and a constant voltage of 5 kV. Nitrogen gas with a flow rate of 150 mL/min was used as the drift gas. Each spectrum was scanned an average of 12 times. The instrument was standardized by the linear retention index (RI) of each volatile compound by using the n-ketones of C4–C9 (Sinopharm Chemical Reagent Beijing Co., Ltd., Beijing, China). The qualitative analysis of volatile compounds was performed by comparing RI and the time required for the ions to reach the collector through the drift tube (drift time, in milliseconds) of the standard from the GC-IMS NIST 11 library. The volatile compounds were quantitatively analyzed based on the peak intensity in HS-GC-IMS, and the content of volatile compounds was directly proportional to the peak intensity.

### 2.4. Data Analysis for GC-IMS

Data from volatile compounds in *T. matsutake* samples were acquired and processed using Laboratory Analytical Viewer (LAV) analysis software and Library Search qualitative software (G.A.S., Dortmund, Germany).

LAV software was used to view the analytical spectrum, where each dot represents a volatile compound. A reporter plugin was directly used to compare the spectral differences between samples (two-dimensional top view and three-dimensional view). A gallery plot plugin was used to compare fingerprints, and visually and quantitatively compare the differences in volatile organic compounds among different samples. A dynamic PCA plugin was used for dynamic principal component analysis and clustering analysis of the samples, and to quickly determine the types of unknown samples. GC-IMS Library Search is an application software that qualitatively analyzes substances in the NIST 11 library and IMS database.

## 3. Results and Discussion

### 3.1. Visual Topographic Plot Comparison

**Figure 1:**
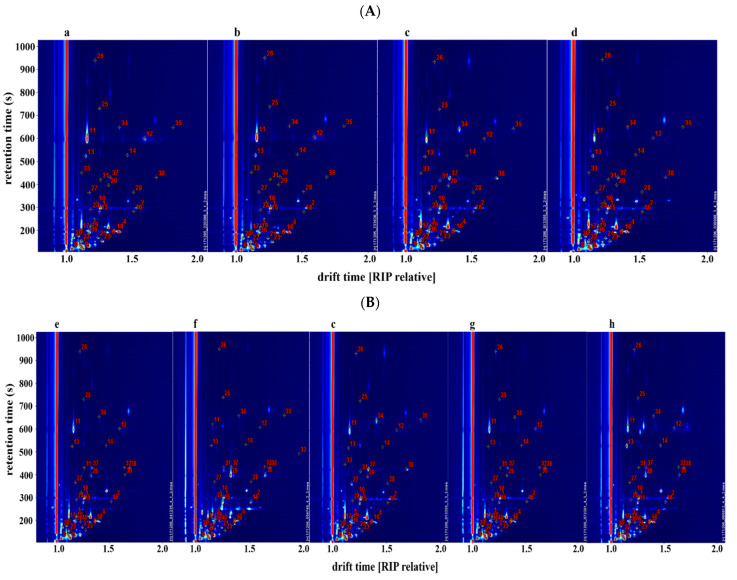
Topographic plots of GC–IMS spectra with the selected markers obtained from *T. matsutake* samples at different frying temperatures (**A**) and frying times (**B**): (**a**) 100 °C, 80 s; (**b**) 120 °C, 80 s; (**c**) 160 °C, 80 s; (**d**) 200 °C, 80 s; (**e**) 160 °C, 40 s; (**f**) 160 °C, 60 s; (**g**) 160 °C, 100 s; and (**h**) 160 °C, 120 s. The numbers correspond to the identified signals.

Figure 1 shows HS-GC-IMS topographical plots of fried samples under different temperatures and times. The *Y*-axis represents the retention time of volatile compounds during GC separation and the *X*-axis represents the drift time of volatile compounds relative to the reactive ion peak during IMS separation. The ion migration time and reactive ion peak (RIP) position were normalized. The total headspace compounds of the samples were displayed throughout the entire spectrum, and each point on the right of the RIP represents a volatile compound extracted from the samples. The color of the signals represents the signal intensity of the compounds. Topographic plots of *T. matsutake* samples from different processing temperatures and times of processing were quite similar; nevertheless, after an intensive visual inspection, features between different categories were found. As shown in Figure 1, most signals were in a 2D spectral region from 1.0 to 1.7 s of drift time and from 0 to 600 s of retention time, whereas few signals were in the retention time range of 600–1000 s. The number of signals in 2D spectral was almost the same. However, the signal intensity changed (increased or decreased). A previous study demonstrated that the low-molecular volatile compounds presented the highest concentration levels and eluted from 100 to 200 s. In addition, the polar volatile compounds showed the lowest concentrations [16]. These results showed that the kinds of volatile compounds in the samples were similar, but the concentrations of volatile compounds changed with the prolonging of frying temperatures and times.

### 3.2. Compound Identification

After analyzing *T. matsutake* samples under different processing conditions, a total of 40 typical target signals from topographic plots (Figure 1) were tentatively identified by comparing the features’ retention and drift times with those of the individual standards’ ion signals. They were confirmed by the commercial GC-IMS library and represented by different numbers (1–40). The identified compounds are listed in Table 1, including the compound name, CAS number, molecular formula, molecular weight, retention time, retention index, and drift time. In addition, other substances with signals that could not be determined were not listed. The tentatively identified volatile compounds in fried *T. matsutake* included six ketones, seven aldehydes, eight alcohols, two esters, and one heterocyclic compound. It has been detected that one analyte might produce multiple signals (protonated monomers or even proton-bound dimers). It has been reported that the formation of dimers or trimers is related to the high proton affinity or high concentration of the compounds in the analytes, and the compounds with high concentration could accelerate the combination of neutral molecules and proton molecules to form dimers [18].

### 3.3. Effects of Different Processing Conditions on Volatile Compounds in T. matsutake

**Figure 2:**
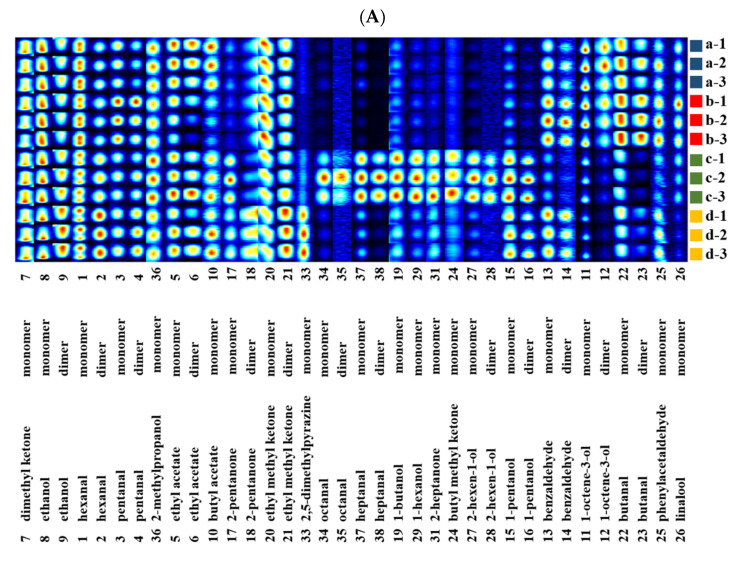

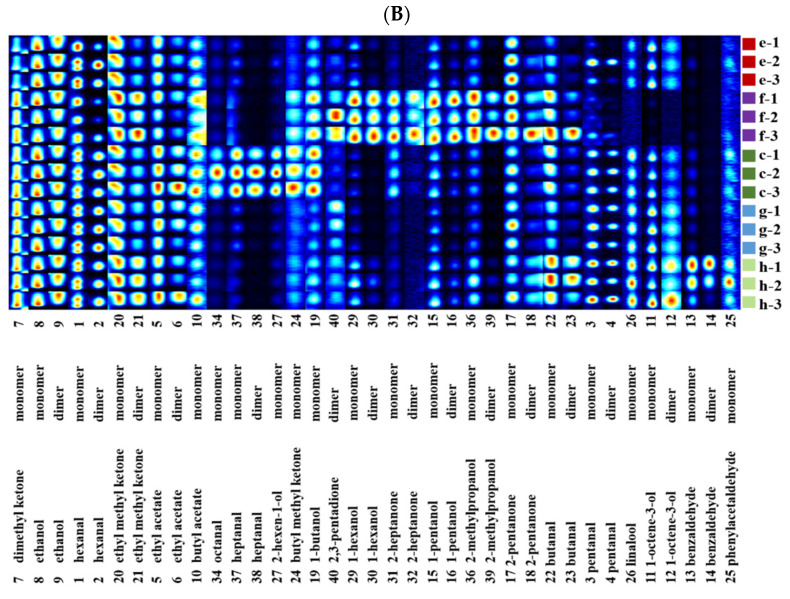
Gallery plot of the selected signal peak areas obtained from *T. matsutake* samples at different frying temperatures (**A**) and frying times (**B**): (**a**) 100 °C, 80 s; (**b**) 120 °C, 80 s; (**c**) 160 °C, 80 s; (**d**) 200 °C, 80 s; (**e**) 160 °C,40 s; (**f**) 160 °C, 60 s; (**g**) 160 °C, 100 s; and (**h**) 160 °C, 120 s.

**Figure 3:**
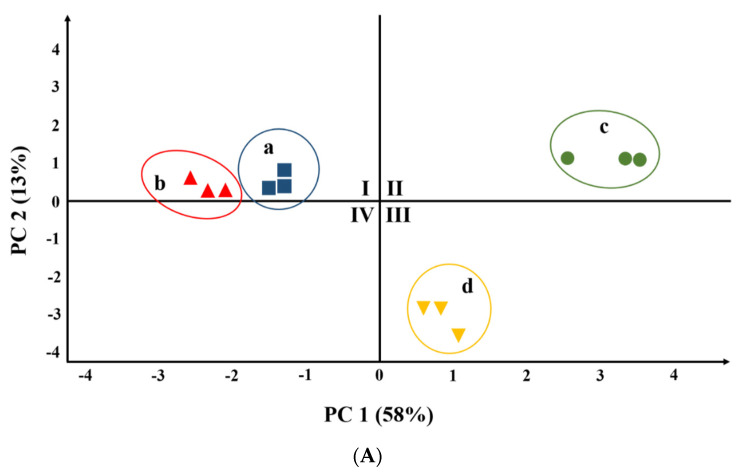

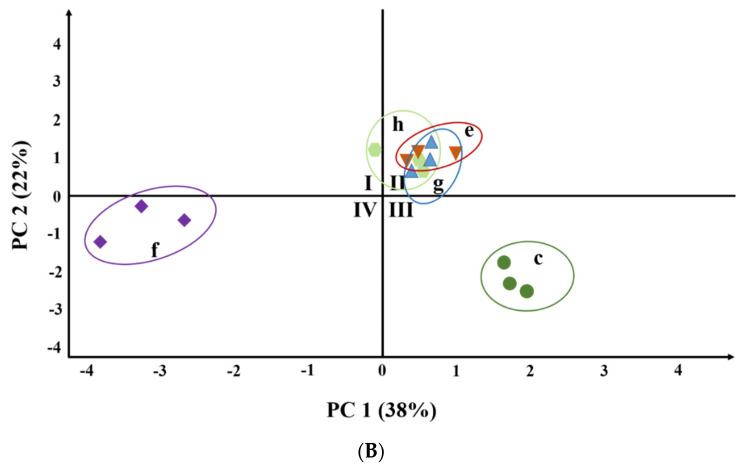
Principal component analysis based on the signal intensity obtained from *T. matsutake* samples at different frying temperatures (**A**) and frying times (**B**): (**a**) 100 °C, 80 s; (**b**) 120 °C, 80 s; (**c**) 160 °C, 80 s; (**d**) 200 °C, 80 s; (**e**) 160 °C,40 s; (**f**) 160 °C, 60 s; (**g**) 160 °C, 100 s; and (**h**) 160 °C, 120 s.

The information about fingerprints was obtained from the signal intensities of all compounds in the GC-IMS topographic plot. The appreciable visual plots were selected and listed together by gallery plot for intuitive comparison (Figure 2 and Figure 3). Each row represents a sample and each column signifies a signal peak. The colors represent the content of volatile compounds. The brighter the color, the higher the content. Accordingly, the differences of non-target volatile compounds in the *T. matsutake* samples under different processing conditions were observed.

By comparing the intensity of the spot, the flavor changes of *T. matsutake* under different processing conditions were determined (increased, decreased, disappeared, or fluctuated). The results in Figure 2 show that the content of volatile compounds in the fried *T. matsutake* samples varied greatly with the increase in frying temperature and time. As the frying temperature increased, the concentration of some volatile compounds such as 2-pentanone, octanal, heptanal, 2-heptanone, 1-hexanol, 2-hexenol, 1-butanol, and butyl methyl ketone increased or newly formed, whereas the concentration of some volatile compounds such as 1-octene-3-ol, benzaldehyde, butanal, phenylacetaldehyde, and linalool decreased gradually. In addition, the *T. matsutake* samples fried at 160 °C contained many specific volatile organic compounds, which indicates that the samples have a special flavor at a frying temperature of 160 °C. Therefore, we further studied the changes of volatile compounds in *T. matsutake* samples at 160 °C with different frying times. It can be seen from Figure 2B that *T. matsutake* fried at 160 °C had different special flavors when heated for 60 s, 80 s, and 120 s. When fried for 40 s, there were many specific compounds, including 2-methyl propanol, 2-pentanone, butanal, butyl methyl ketone, 1-butanol, 2,3-pentadione, 1-hexanol, 2-heptanone, and 1-pentanol. The most prominent volatile compounds were octanal, heptanal, 2-hexenol, butyl methyl ketone, and 1-butanol when fried for 80 s. However, when fried for 120 s, 2-pentanone, butanal, pentanal, linalool, 1-octene-3-ol, benzaldehyde, and phenylacetaldehyde showed clear differences compared with other samples.

The delicious and unique flavors of fried *T. matsutake* are mainly formed by numerous chemical reactions of volatile compounds during frying. The Maillard reaction, interaction of proteins or amino acids with oxidized lipids, oxidation and degradation of lipids, and degradation of long-chain compounds during thermal processing may form some volatile flavor compounds [19,20]. The Maillard reaction is a complex reaction process to produce aldehydes, pyrazines, ketones, furans, and other flavor compounds; heating temperature, reaction time, water content, chemical composition, and other factors affect the Maillard reaction [21]. With an increase in temperature, the contents of some aldehydes decreased first and then increased gradually, and the contents of some aldehydes increased or even newly formed. It was reported that some of the higher activity intermediates (dehydrogenated reductones) in the Maillard reaction were unstable, which easily led to deamination and decarboxylation reactions of amino acids and the formation of aldehydes [22]. Most aldehydes had fruity, fatty, and nutty flavors and were mainly produced by lipid oxidation, which has a lower odor threshold concentration [23]. With the increase in heating temperature and time, the contents of identified esters remained unchanged. Esters play an important role in the formation of the overall flavor of T. matsutake, which can be formed by the interaction of free fatty acids and alcohols produced by the oxidation of fats [24], resulting in a flavor with a variety of fruity characteristics [25]. A previous study reported the production of esters and hydrocarbons, which may be due to ester degradation and the cleavage reaction of alkoxy radicals induced by the heating and baking process [26,27].

A group of C8 compounds, including 1-octen-3-ol (mushroom), (E)-2-octen-1-ol (mushroom), 3-octanol (mushroom and moss), 1-octanol (chemical and sweet), 3-octanone (herb), and (E)-2-octenal (green and sweet), have been reported to be typical volatile compounds in edible mushrooms, and are major contributors to the unique flavor of mushrooms [10]. A previous study reported that 1-octene-3-ol is the main volatile compound found in mushrooms and is known as mushroom alcohol [28]. At different temperatures, the decrease of C8 volatile compounds dominated the changes to important volatile compounds. This result may be affected by the chemical volatilization and peroxidation of polyunsaturated fatty acids [27]. The main biosynthetic pathway of C8 compounds in edible fungi is formed by the enzymatic oxidative cleavage of linoleic acids or linolenic acids under the action of enzymes such as lipoxygenase (LOX) and hydroperoxide lyase [29]. Guo et al. [10] studied the water dynamics of *T. matsutake* during heat treatment and proved that the destruction of the cell structure led to a decrease in the level of most C8 compounds.

Studies have shown that alkanes and alcohols can transform each other, alter the sensory properties of foods, and play a role in the reconciliation and complementation of the flavor of *T. matsutake* [30]. It was observed that 2,3-pentanedione and 2,5-dimethyl pyrazine changed with the increase in frying temperature and time. Ketones are the products of the oxidation of alcohols or the decomposition of esters. However, due to the high odor threshold concentration of ketones, their overall contribution to the flavor of *T. matsutake* is not great [27]. Heterocyclic compounds (pyrazines) are an important source of the unique aromatic odor of T. matsutake, which has high odor intensity with nutty and roasted flavors [30]. During the heating and baking process, the six-membered ring structure of pyrazines is destroyed, resulting in the production of nitrogen-containing olefins and leading to an increase in hydrocarbon compounds [31].

### 3.4. *T. matsutake* Discrimination by Chemometric Methods

Principal component analysis (PCA) is a common chemometric method that can reduce multiple indicators into several comprehensive indicators, simplify data, and reveal the relationship between variables based on making full use of most of the original variable information [32]. To obtain an overview of the similarities and differences, PCA was carried out to highlight the differences in volatile compounds among *T. matsutake* samples by signal intensity. As shown in Figure 3, *T. matsutake* samples from different processing conditions were well separated and clustered according to their signal intensity in a relatively independent space in the PCA score plot.

Figure 3A shows that the *T. matsutake* samples fried at different temperatures for 80 s could be distinguished well in PC1, and there was a great difference between samples c (160 °C, 80 s) and d (200 °C, 80 s). Figure 3B shows that samples c (160 °C, 80 s) and f (160 °C, 60 s) differed greatly from other samples with different specific flavors. Moreover, there were great differences between samples c and f. The volatile compounds in samples e (160 °C, 40 s), g (160 °C, 100 s), and h (160 °C, 120 s) were not distinguished. Combining the results of Figure 3A,B shows that samples heated at different temperatures were more decentralized than those under different heating times. This indicates that the heating temperature was a more important factor than the heating time in influencing the change of volatile compounds in the *T. matsutake* samples.

## 4. Conclusions

In this paper, the advantages of HS-GC-IMS rapid qualitative analysis combined with PCA were used to analyze the changes to volatile compounds in *T. matsutake* samples under different processing conditions and the flavor fingerprints were established. A total of 24 volatile compounds from topographic plots were identified by HS-GC-IMS. The tentatively identified substances in fried *T. matsutake* included six ketones, seven aldehydes, eight alcohols, two esters, and one heterocyclic compound. Based on topographic plots and fingerprints, it was observed that the change in the main flavor compounds in *T. matsutake* was based on the thermal decomposition and a series of chemical reactions of C8 compounds such as alcohols. Other compounds such as esters, alkanes, and aldehydes, including methyl cinnamate, pentadecane, hexadecane, furfural, 2-pentylfuran, and 1-octen-3-one, played a role in the reconciliation and complementation of the flavor of *T. matsutake* samples. PCA results indicated that the samples heated at different temperatures were more decentralized than those under different heating times, and the heating temperature was more important than the heating time in influencing the change of volatile compounds in the *T. matsutake* samples. This study confirmed the potential of HS-GC-IMS combined with PCA as a reliable analytical screening technique to identify and classify the volatile compounds of *T. matsutake* quickly and sensitively. The above results can provide a theoretical basis for the quality control and change mechanism of flavor in the processing of traditional edible fungi products.

## Figures and Tables

**Table 1:** GC-IMS integration parameters of volatile compounds in *T. matsutake* samples under different processing conditions.

No.	Compound	CAS#	Formula	MW	RT ^1^	RI ^2^	DT ^3^	Identification Approach
1	hexanal	66-25-1	C_6_H_12_O	100.2	300.802	798.5	1.2642	RI, DT
2	hexanal dimer	66-25-1	C_6_H_12_O	100.2	300.802	798.5	1.564	RI, DT
3	pentanal	110-62-3	C_5_H_10_O	86.1	218.794	697.5	1.1929	RI, DT
4	pentanal dimer	110-62-3	C_5_H_10_O	86.1	217.721	695.8	1.4219	RI, DT
5	ethyl acetate	141-78-6	C_4_H_8_O_2_	88.1	176.719	619.9	1.0978	RI, DT
6	ethyl acetate dimer	141-78-6	C_4_H_8_O_2_	88.1	176.719	619.9	1.338	RI, DT
7	dimethyl ketone	67-64-1	C_3_H_6_O	58.1	133.865	497.2	1.1151	RI, DT
8	ethanol	64-17-5	C_2_H_6_O	46.1	122.644	449.6	1.0405	RI, DT
9	ethanol dimer	64-17-5	C_2_H_6_O	46.1	122.451	448.7	1.1298	RI, DT
10	butyl acetate	123-86-4	C_6_H_12_O_2_	116.2	318.265	815.1	1.2373	RI, DT
11	1-octene-3-ol	3391-86-4	C_8_H_16_O	128.2	600.2	987.2	1.1607	RI, DT
12	1-octene-3-ol dimer	3391-86-4	C_8_H_16_O	128.2	597.74	986.1	1.6013	RI, DT
13	benzaldehyde	100-52-7	C_7_H_6_O	106.1	522.824	951.6	1.1511	RI, DT
14	benzaldehyde dimer	100-52-7	C_7_H_6_O	106.1	522.383	951.4	1.4735	RI, DT
15	1-pentanol	71-41-0	C_5_H_12_O	88.1	276.177	772.6	1.2525	RI, DT
16	1-pentanol dimer	71-41-0	C_5_H_12_O	88.1	275.492	771.9	1.5139	RI, DT
17	2-pentanone	107-87-9	C_5_H_10_O	86.1	198.516	663.4	1.1164	RI, DT
18	2-pentanone dimer	107-87-9	C_5_H_10_O	86.1	197.023	660.7	1.3741	RI, DT
19	1-butanol	71-36-3	C_4_H_10_O	74.1	203.225	671.8	1.183	RI, DT
20	ethyl methyl ketone	78-93-3	C_4_H_8_O	72.1	167.915	599.6	1.0598	RI, DT
21	ethyl methyl ketone dimer	78-93-3	C_4_H_8_O	72.1	168.037	599.9	1.2463	RI, DT
22	butanal	123-72-8	C_4_H_8_O	72.1	152.648	559.3	1.1156	RI, DT
23	butanal dimer	123-72-8	C_4_H_8_O	72.1	153.561	562.0	1.2854	RI, DT
24	butyl methyl ketone	591-78-6	C_6_H_12_O	100.2	289.927	787.4	1.1898	RI, DT
25	phenylacetaldehyde	122-78-1	C_8_H_8_O	120.2	727.771	1035.8	1.2575	RI, DT
26	linalool	78-70-6	C_10_H_18_O	154.3	929.821	1096.1	1.2226	RI, DT
27	2-hexen-1-ol	2305-21-7	C_6_H_12_O	100.2	361.404	851.6	1.1823	RI, DT
28	2-hexen-1-ol dimer	2305-21-7	C_6_H_12_O	100.2	361.404	851.6	1.5179	RI, DT
29	1-hexanol	111-27-3	C_6_H_14_O	102.2	394.267	875.9	1.3278	RI, DT
30	1-hexanol dimer	111-27-3	C_6_H_14_O	102.2	392.952	875.0	1.6465	RI, DT
31	2-heptanone	110-43-0	C_7_H_14_O	114.2	416.544	891.0	1.2622	RI, DT
32	2-heptanone dimer	110-43-0	C_7_H_14_O	114.2	416.023	890.7	1.6307	RI, DT
33	2,5-dimethylpyrazine	123-32-0	C_6_H_8_N_2_	108.1	446.298	909.7	1.1201	RI, DT
34	octanal	124-13-0	C_8_H_16_O	128.2	640.818	1003.8	1.4101	RI, DT
35	octanal dimer	124-13-0	C_8_H_16_O	128.2	642.699	1004.6	1.8248	RI, DT
36	2-methylpropanol	78-83-1	C_4_H_10_O	74.1	183.978	635.4	1.1713	RI, DT
37	heptanal	111-71-7	C_7_H_14_O	114.2	428.749	898.9	1.3363	RI, DT
38	heptanal dimer	111-71-7	C_7_H_14_O	114.2	429.24	899.2	1.6973	RI, DT
39	2-methylpropanol dimer	78-83-1	C_4_H_10_O	74.1	183.087	633.5	1.3627	RI, DT
40	2,3-pentadione	600-14-6	C_5_H_8_O_2_	100.1	196.585	654.4	1.2204	RI, DT

^1^ Represents the retention time in the capillary GC column. ^2^ Represents the retention index calculated using n-ketone C_4_-C_9_ as the external standard in the FS-SE-54-CB column. ^3^ Represents the drift time in the drift tube.

## Data Availability

The data showed in this study are contained within the article.
